# Assessment System and Optimization of the Thermal Extraction Methods for Extracellular Polymeric Substances (EPS) from *Microcystis*

**DOI:** 10.3390/microorganisms14010116

**Published:** 2026-01-05

**Authors:** Yafei Cui, Sheng Zhang, Pengbo Zhao, Jingyuan Cui, Shuwei Song, Yao Qu, Haiping Zhang, Dong Ma

**Affiliations:** 1 Academy of Forensic Science, Shanghai 200063, China; cuiyf@ssfjd.cn; 2 Department of Environmental Science, College of Environmental Science and Engineering, Tongji University, Shanghai 200092, China; 3 Institute of Water Sciences, Zhejiang University of Water Resources and Electric Power, Hangzhou 310018, China; 4 School of Marxism, Tongji University, Shanghai 200092, China

**Keywords:** quantitative assessment system, bound extracellular polymeric substances (B-EPS), *Microcystis*, extraction methods

## Abstract

Extracellular polymeric substances (EPS) play crucial roles in the growth and survival of microorganisms. However, the lack of effective evaluation for extraction methods has limited further investigations and applications of EPS. This study established a quantitative assessment system for algal EPS thermal extraction methods based on extraction yield, cell integrity, and EPS chemical properties. An extraction efficiency parameter (ε) was introduced to quantify the relationship between EPS yield and cell rupture. Thermal treatment proved to be an effective approach for algal EPS extraction. Using the proposed evaluation system, the extraction methods for EPS of *Microcystis* were compared and optimized, including the following treatments: NaOH, NaCl, and buffer solutions (borate, phosphate, Tris-HCl). The results demonstrated that heating at 55 °C for 30 min with borate buffer achieved the highest extraction efficiency for EPS, with an ε value of 11.06 ± 1.13. In contrast, NaOH treatment at 60 °C for 30 min resulted in 30.4% cell rupture and the lowest ε value (9.7 ± 0.81). Furthermore, the modeled cell rupture rates aligned with flow cytometry and three-dimensional fluorescence spectroscopy analyses. The EPS extraction evaluation system developed in this study was empirically validated as a robust tool for optimizing extraction protocols for algal EPS.

## 1. Introduction

EPS play crucial roles in the growth and survival of microorganisms. EPS are involved in surface bloom formation of *Microcystis* and nitrogen cycling [1,2,3]. Moreover, EPS can increase the tolerance of algal cells to toxicants (heavy metal, dehydration, microplastics (MPs) and nanoplastics (NPs)) through protective mechanisms (the increment of secretion, sequestration of toxicants by the EPS matrix, sacrificial interaction of EPS with toxicants) [4,5,6,7]. Moreover, EPS can be served as carbon/energy sources for organisms in starvation via nutrient exchanges with heterotrophic bacteria (HB) growing in the micro-environment created by EPS [8,9]. Therefore, it is essential to extract EPS from microalgal cells for further quantitative analysis and fundamental scientific studies.

EPS matrices and cells are integrated via van der Waal forces, electrostatic interactions, hydrogen bonds, and hydrophobic interactions [6,10]. EPS can be classified into soluble EPS (S-EPS) and B-EPS based on their binding affinity to the cell surface [11]. S-EPS exhibit weak interactions with cellular membranes, allowing diffusion into the extracellular medium, whereas B-EPS demonstrate either loose or tight adhesion to cell surfaces. For S-EPS, high-speed centrifugation is the most widely adopted and efficient method. Hydrogen bonding and van der Waal forces in EPS matrices can be effectively reduced at higher temperatures, resulting in EPS solubilization. Therefore, thermal methods are frequently employed in EPS extraction from *Microcystis* [12]. However, a standard thermal method is not available to extract microalgal B-EPS, which restricts the comparison among different studies.

The operational conditions vary, and the results differ significantly in different reports. Numerous studies have conducted comparative analyses of the available EPS thermal extraction methods. Comparisons of the available methods have been performed in many studies. For example, methods such as heating, ultrasound-assisted extraction, and treatment with ethylenediaminetetraacetic acid (EDTA), cation exchange resin (CER), sodium hydroxide (NaOH), or formaldehyde were compared for extracting B-EPS from cyanobacterial blooms, among which heating at 60 °C for 30 min was reported to be the best method to isolate B-EPS [13]. Liu et al. used an alkaline extraction method with NaOH solution (pH = 10) [14]. Xu et al. suggested that heating a 0.05% NaCl (*w*/*v*) solution at 60 °C for 30 min is the most effective method [15]. Duan et al. found that buffer solutions can maintain algal cell integrity during thermal extraction, and 65 °C and 30 min in boric acid buffer (pH = 8.5) were the optimal parameters [12]. However, the lack of standardized evaluation methods critically impedes systematic review and cross-study comparison. Developing a systematic, quantitative assessment system is, therefore, imperative to enable rigorous method comparison.

Conventionally, EPS extraction methods are assessed based on the following key criteria. The ideal standard for EPS extraction methodology necessitates simultaneous fulfillment of three interdependent criteria: 1. maximized extraction field; 2. avoiding cell membrane rupture; 3. maintaining chemical properties [16,17]. Therefore, cell integrity or cell lysis must be evaluated during EPS extraction.

This study established a B-EPS extraction efficiency evaluation system, integrating three key metrics: EPS yield, algal cell integrity, and EPS property analysis. Based on the system, the EPS thermal extraction protocol was systematically optimized, and the results were compared with existing protocols available, evaluating both extraction efficiency, cell integrity, and chemical properties.

## 2. Materials and Methods

### 2.1. Algae

*Microcystis aeruginosa* FACHB-912 was purchased from the Freshwater Algae Culture Collection of the Institute of Hydrobiology, Chinese Academy of Sciences, Wuhan, China. Algae cells were cultivated in BG-11 medium in a light incubator (HGZ-250, Huitai Co., Shanghai, China) at 25 °C and a light intensity of 30 μmol photons m^−2^ s^−1^ under 12 h:12 h light–dark cycle. All cultures were harvested in the early stationary phase.

### 2.2. Extracellular Polymeric Substances (EPS) Extraction

In this study, comparative experiments involved five thermal extraction methods for bound EPS (B-EPS), including NaOH, NaCl, phosphate buffer, borate buffer, and Tris-HCl buffer. These buffers are commonly used as pH and osmotic pressure stabilizers in microalgae/bacterial EPS extraction [12]. Comparisons among extraction methods were conducted under gradient temperature and time conditions. Detailed extraction procedures are described in Appendix A.

### 2.3. Biochemical Composition Analysis

The total organic carbon (TOC) was measured using a TOC analyzer (Shimadzu, TOC-L CPH, Kyoto, Japan). The content of total polysaccharides in the supernatant was quantified using the anthrone-sulfuric acid method, with glucose as the standard [18]. Protein contents were determined using the Coomassie Blue method, with bovine serum albumin (BSA) as the standard [19]. The nucleic acid contents were measured using the diphenylamine method [20], with calf thymus DNA (D8020, Solarbio, Beijing, China) as the standard.

### 2.4. Flow Cytometry (FCM) Analysis for Cell Integrity Test

A FACSVerse flow cytometer (BD Biosciences, San Jose, CA, USA) was used to evaluate the integrity of the extracted algal cells. The algal cells collected through centrifugation were washed with phosphate buffered saline (PBS) and stained with the fluorescent dye SYBR Green I. SYBR Green I stains the nucleic acid to form complexes in dead cells that have permeable membranes. The complexes were excited by the blue light laser with a wavelength of 488 nm, which produced a fluorescent emission at 520 nm. The forward scatter (FSC) and fluorescent channel (fluorescein isothiocyanate (FITC): 510–550 nm) were determined for each algal cell. FSC is an indicator of cell size and shape, and FITC indicates the relative nucleic acid content. The data analysis and graphical outputs were performed with FlowJo 7.6.1 software (Treestar, Inc., Chico, CA, USA).

### 2.5. Microscopic Analysis and Quantification of Cell Disruption

Morphological characterization of treated cultures was performed using a Motic BA310 microscope (Motic, Xiamen, China) equipped with a Moticam Pro 285A camera (Motic, Xiamen, China). Digital images were acquired and analyzed using Motic Images Advanced 3.2 software (Motic, Xiamen, China). To quantify cell lysis, cell counts were performed using a hemocytometer (Olympus CX31 microscope, Olympus, Tokyo, Japan). Each sample was analyzed in triplicate (n ≥ 6), and the mean value was calculated. The cell lysis rate (CL, %) was determined using the formula CL (%) = (C0 − Cl)/C0 × 100%, where C0 denotes the initial cell concentration and C1 represents the post-treatment cell concentration.

### 2.6. Particle Size Distribution (PSD) Analysis

To determine the impact of thermal treatment on cell size, a laser diffraction particle size analyzer (Mastersizer 2000, Malvern Panalytical, Malvern, UK) was utilized to measure the PSD of the samples. Milli-Q water with a refractive index of 1.33 was used as the dispersant. For all the samples, a refractive index of 1.40 and a light absorption index of 0.1 were used [21].

### 2.7. Three-Dimensional Excitation-Emission Matrix (3D-EEM) Fluorescence Spectroscopy

3D-EEM fluorescence spectroscopy was employed to analyze compositional changes in B-EPS. Spectral measurements were conducted using a Hitachi F-7000 FL luminescence spectrophotometer (Hitachi, Tokyo, Japan). The 3D-EEM spectra were collected subsequent scanning emission spectra from 220 to 550 nm at 5 nm increments by varying the excitation wavelength from 200 to 450 nm at 5 nm sampling intervals under a scan speed of 1200 nm/min. The excitation and emission slits were maintained at 5 nm, and the spectrum of ultrapure water was measured as the blank. The resulting 3D-EEM spectra were analyzed using the software Origin 2020 (OriginLab, Northampton, MA, USA) and represented in the form of a contour map.

### 2.8. EPS Extraction Efficiency Evaluation

The extraction of EPS adheres to three fundamental criteria: high yields of EPS, low cell lysis EPS, and chemical properties. In prior research, methodologies for isolating EPS from microalgae, activated sludge, and biofilms were predominantly assessed by quantifying the polysaccharide and protein content as the yield of EPS, while the cell lysis was evaluated based on the nucleic acid content [22,23]. In this study, we employed the dimensionless extraction efficiency parameter (ε) to quantitatively evaluate EPS extraction efficiency from *Microcystis*. This parameter is defined as the ratio of extracted EPS content to the mass of released intracellular organic matter per unit biomass. The mathematical formulation of this optimized model is detailed below.

To further characterize the process of EPS extraction and release of intracellular organic matter during the extraction process, several parameters and equations were set based on the following assumptions:

(1) The treated *Microcystis* cells have two states of cells: intact cells and broken cells, where the proportion of broken cells is α.
(2) Since *Microcystis* is prokaryotic and does not contain a nucleus, almost all nucleic acids are present in the cytoplasm.
(3) Intracellular organic constituents (including polysaccharides, proteins, nucleic acids, and related biomolecules) exhibit homogeneous spatial distribution patterns within individual algal cells. [*TOC_E_*], [*TOC_I_*], and *C_N_* are the extracellular total organic matter content, the intracellular total organic matter content, and the average content of nucleic acids in individual *Microcystis* cells, respectively. In summary, the measured TOC and nucleic acid expression can be obtained.

(1) $$\partial_{1}\left[Nc\right]=\alpha nC_{N}$$

(2) $$\left[TOC\right]=\varphi n\left[{TOC}_{E}\right]+\alpha n\left[{TOC}_{I}\right]$$

where *∂*_1_ is the proportion of intracellular DNA yields to the total DNA yields, including both intracellular and extracellular DNA and within the range of 96.5–98.7% [13], which was determined to be 97% in the study; [*Nc*] is the measured content of nucleic acids; α is the proportion of ruptured algal cells; n is the number of algal cells; [*TOC*] is the measured content of TOC; *φ* is the proportion of extracted EPS; and *ε* is the yield of extracted EPS per unit mass of released intracellular organic matter. To evaluate the extraction efficiency by considering the yield of EPS and the interference of intracellular organic compounds, the parameter *ε* was defined in this study as the ratio of the extracted EPS content to the yield of released intracellular organic compounds. It can be expressed by Equation (3):

(3) $$\varepsilon =\frac{\varphi \left[{TOC}_{E}\right]}{\alpha \left[{TOC}_{I}\right]}$$

We can determine the expressions of φ and ε through Equations (1)–(3):

(4) $$\varphi =\frac{\left[TOC\right]C_{N}{-\partial }_{1}\left[{TOC}_{I}\right]\left[Nc\right]}{\left[{TOC}_{E}\right]\left[Nc\right]}$$

(5) $$\varepsilon =\frac{\left[TOC\right]C_{N}{-\partial }_{1}\left[{TOC}_{I}\right]\left[Nc\right]}{\alpha \left[Nc\right]\left[{TOC}_{I}\right]}$$

The values of [*TOC_E_*], [*TOC_I_*], and *C_N_* were determined with the following method. The algal cells were treated with an ultrasonic cell disruptor (Bilang1500Y, Shanghai, China) for 300s at 300 W. The algal cells were assumed to completely release intracellular organic matter under the conditions. As a result, the value of [Nc] was measured to be 4.28 mg/L. [*TOC_E_*] and [*TOC_I_*] are the total contents of extracellular or intracellular TOC in a tube of microalgal sample, which are 5.11 mg/L and 4.12 mg/L, respectively.

### 2.9. Statistical Analysis

Each treatment was conducted in triplicate. Data were presented as mean ± standard deviation. After examining their homoscedasticity, significant differences among the results of different treatments were analyzed using one-way ANOVA. All statistical analyses were conducted in Origin (OriginLab, Northampton, MA, USA). The significance level for comparative purposes was defined as a *p*-value < 0.05.

## 3. Results and Discussion

### 3.1. Evaluation System for EPS Extraction Methods

**Figure 1:**
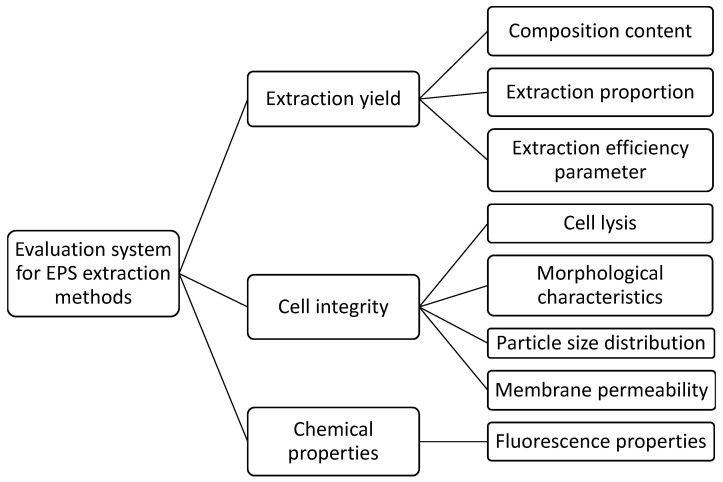
Frame diagram of evaluation system for EPS extraction methods.

An evaluation system for EPS extraction methods based on EPS extraction principles was established, considering three key dimensions: EPS extraction yield, cellular integrity, and EPS properties (Figure 1). A quantitative relationship between EPS extraction yield and algal cell disruption was developed—the extraction efficiency parameter (*ε*) was introduced to evaluate the EPS extraction efficiency from *Microcystis*. *ε* is defined as the amount of EPS extracted per unit mass of released intracellular organic matter. A preliminary assessment of cellular integrity was conducted through microscopic examination and particle size distribution (PSD) analysis to monitor morphological changes and particle size variations before and after extraction. The fluorescent properties of the extracted EPS were also characterized. Furthermore, flow cytometry (FCM) was employed to provide a detailed investigation of indicators reflecting cellular integrity, including cell membrane permeability and cell size. This approach enhances the quantitative evaluation system for EPS extraction methods.

### 3.2. Biochemical Compositions of B-EPS Extracted by Different Methods and Conditions

**Figure 2:**
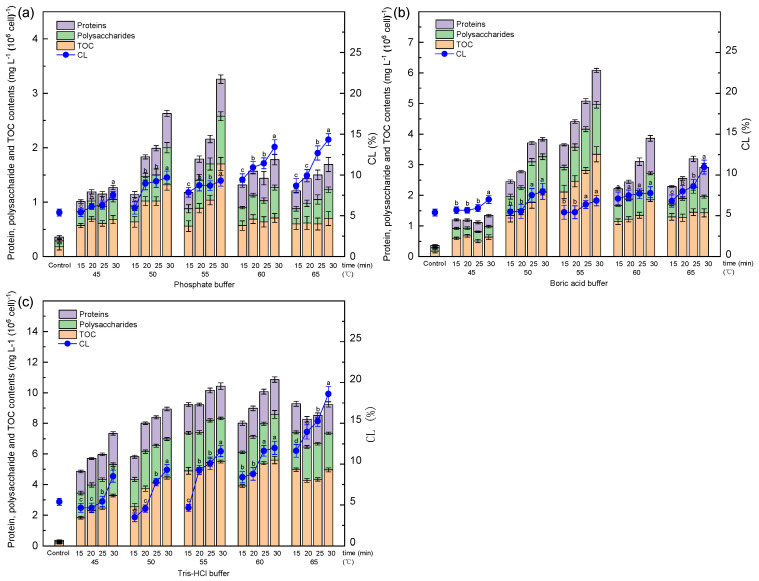
Biochemical compositions of B-EPS extracted from *Microcystis* via thermal buffer solution methods: (**a**) phosphate buffer, (**b**) boric acid buffer, (**c**) Tris-HCl buffer. Different letters indicate significance of CL between different treatment times (*p* < 0.05).

The contents of TOC, proteins, and polysaccharides of B-EPS extracted from *Microcystis* by different methods and conditions are shown in Figure 2. The yield of B-EPS showed higher divergence with different extraction methods. At lower temperatures (<50 °C), no significant changes were observed in the content of organic compounds, represented by TOC, polysaccharides, and protein content. With the increase in extraction temperature and time, EPS production shows an increasing trend. Polysaccharides and proteins are the main components of EPS; therefore, the increase in polysaccharide and protein content indicates an increase in the extraction yield of EPS [12].

For phosphate buffer and borate buffer, the yield of EPS of *Microcystis* increased until the heating temperature reached 55 °C and 30 min, and for Tris-HCl buffer, the critical extraction condition was 60 °C and 30 min. The maximum extraction yields of *Microcystis* with different buffer solutions are shown in Table 1. The results show that the optimal extraction methods are different and cannot be generalized. In addition, higher nucleic acid content was observed under relatively harsh extraction conditions than under mild conditions, which means that more cell lysis is caused.

**Figure 3:**
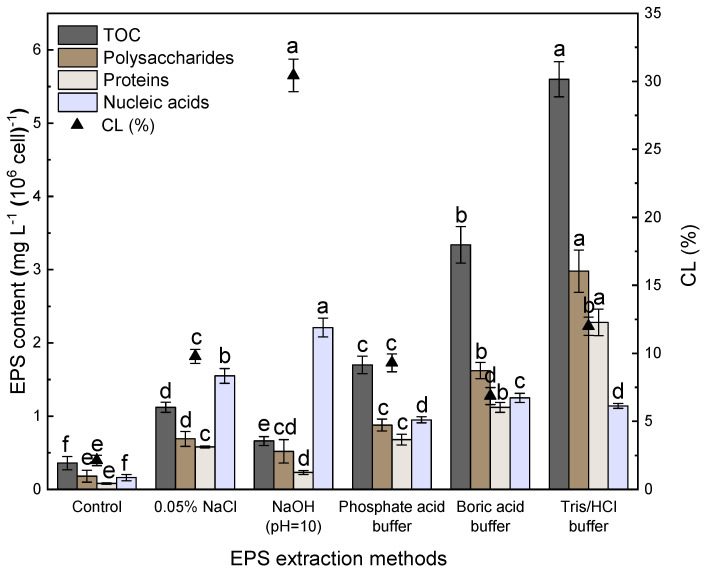
Comparison of different methods for the extraction of EPS from *Microcystis* in terms of EPS yield (total organic carbon (TOC), polysaccharides, proteins, and nucleic acids) and CL (the percentage of cell lysis in the extraction). Error bars represent standard deviation (SD) of triplicate samples. Different letters indicate significance between different treatments (*p* < 0.05).

Compared to the control group, five extraction methods could extract EPS from *Microcystis aeruginosa*, which, however, were different in optimal extraction conditions (Figure 3). EPS yield extracted by thermal treatment with buffer solution (phosphate buffer method; boric acid buffer method; Tris-HCl buffer method) was higher than those extracted by the 0.05% NaCl and NaOH (pH = 10) methods. This indicates that buffer solution treatment method facilitates the extraction of more EPS from *Microcystis*. Specifically, among the buffer solution extraction methods, the maximum EPS yield was obtained with the Tris-HCl buffer treatment, and its TOC, polysaccharide, and protein yields were 3.29, 1.68 times; 3.39, 1.84 times; and 3.35, 2.04 times higher than those of phosphate buffer and boric buffer, respectively. TOC yields extracted by the 0.05% NaCl and NaOH (pH = 10) methods were reduced by 80%, 88%, respectively, while polysaccharide and protein yields were reduced by 77%, 83%; and 75%, 90%, respectively, compared to those of Tris-HCl buffer. This could be attributed to the decrease in EPS extraction and increase in release of cellular inclusions.

Interestingly, NaOH (pH = 10) extraction caused a breakage rate of 30.43%, which was far higher than other methods, but there was no increase in the extracted EPS yield (TOC 0.66 ± 0.06 mg L^−1^, polysaccharides 0.52 ± 0.16 mg L^−1^, proteins 0.23 ± 0.03 mg L^−1^), which was lowest among the five selected methods. NaOH solution leads to the ionization of carboxyl groups, resulting in a strong repulsion and separation between B-EPS and the cells, which lyses the cells and increases cell rupture [13]. However, polysaccharides can decompose and flocculate under alkaline conditions [24], and the copolymerization and degradation of organic matter could lead to a decrease in the yield of B-EPS [25]. The EPS fields extracted by the 0.05% NaCl method (TOC 1.12 ± 0.07 mg L^−1^, polysaccharides 0.69 ± 0.1 mg L^−1^, proteins 0.58 ± 0.01 mg L^−1^) were higher than the NaOH (pH = 10) method, and the CL (9.78% ± 0.52%) was lower than that of the NaOH method. As an osmolality stabilizer, a certain concentration of NaCl can increase the osmotic pressure in the solvent and inhibit the entry of water into the cells, thus reducing the rupture of algal cells during extraction [26]. This mechanism may be responsible for the lower cell lysis rate extracted via the 0.05% NaCl method. As shown in Figure 3, the CL rates of the buffer solution methods (boric acid buffer, phosphate buffer, Tris-HCl buffer) are significantly lower than that of the NaOH (pH = 10) method (*p* < 0.05). It suggests that heating the buffer solution under certain conditions could increase the yield of EPS with lower cell disruption.

### 3.3. Cell Integrity Analysis

Algal cell lysis typically results in cellular debris, causing a reduction in cell size and inducing cell rupture, which can be utilized to monitor cell destruction [25]. In flow cytometry analysis, cell size is usually measured by forward scatter (FSC), while FITC can be used to determine relative cellular nucleic acid content. The P1 and P2 gates indicate the percentage of cell debris and intact cells, respectively. The Q1 and Q2 gates indicate the percentage of cells or cell debris without FL1 fluorescence and cells with fluorescence, respectively (Table 2).

After treatment with 0.05% NaCl solution and NaOH (pH = 10) solution, a lower proportion of intact cells identified by the P2 gate was observed, with values of 69.5% and 50.2%, respectively, while the percentage of cellular debris increased to 30.5% and 49.8%, respectively (Table 2). Compared with the control group, the signal intensity of FSC treated with 0.05% NaCl and NaOH (pH = 10) solution showed only a marginal reduction in cell size, with a decrease from 1.5 × 10^5^ to 0.7 × 10^5^. In contrast, thermal buffer treatments resulted in a higher proportion of intact cells identified by the P2 gate, accounting for >91.9–94.3% (93.2%) of the total pellet, and a low percentage of cellular debris (5.7–8.1%) was detected in all groups as evidenced by the P1 gate. This suggests that the NaOH (pH = 10) method damages *Microcystis* cells or induces cell debris formation, but the thermal treatments with the phosphate buffer, boric acid buffer, and Tris-HCl buffer did not significantly induce cell lysis.

### 3.4. Chemical Characteristics of EPS Extracted from Microcystis

**Figure 4:**
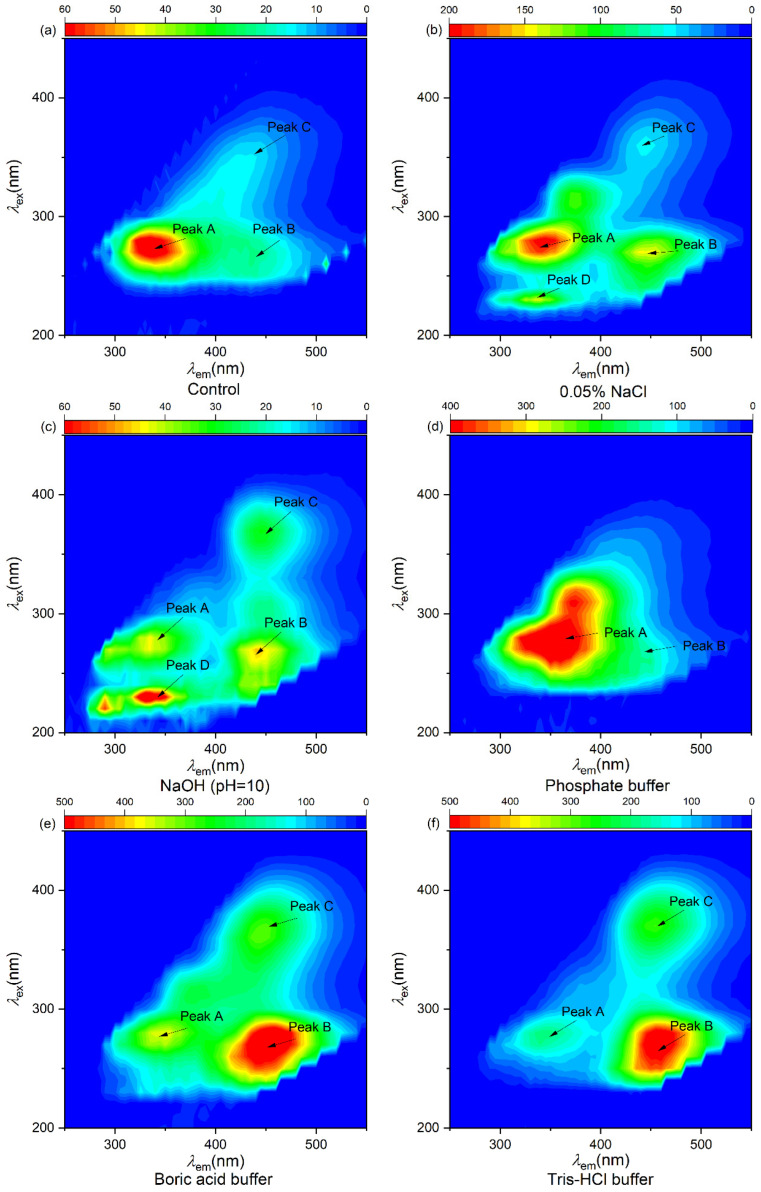
3D-EEM fluorescence spectra of EPS extracted from *Microcystis* via different methods. (**a**) Control, (**b**) 0.05% NaCl (*w*/*v*) solution, (**c**) NaOH (pH = 10) solution, (**d**) phosphate buffer, (**e**) boric acid buffer, (**f**) Tris-HCl buffer. Peaks A, B, C, and D represent tryptophan-like substances, humic acid-like substances, and tyrosine-like substances, respectively.

The chemical characteristics of EPS extracted from *Microcystis* were discussed by the fluorescence peak positions and fluorescence intensity with the 3D-EEM spectra. There were four fluorescence peaks in EPS: peak A (λ_ex_/λ_em_ = 280 nm/335–340 nm, tryptophan-like substances), peak B (λ_ex_/λ_em_ = 230–260 nm/420–450 nm humic acid-like substances) [27], peak C (λ_ex_/λ_em_ = 355–360 nm/455 nm, humic acid-like substances), and peak D (λ_ex_/λ_em_ = 225–230 nm/330–340 nm, tyrosine-like substance) [28] (Figure 4). EPS extracted by 0.05% NaCl (*w*/*v*) and NaOH (pH = 10) solution contained four fluorescence peaks (peak A, B, C, and D) (Figure 4b,c), and EPS extracted by boric acid buffer, Tris-HCl buffer, and control methods contained peak A, peak B, and peak C (Figure 4e,f), while EPS extracted by phosphate buffer only contained peak A and peak B (Figure 4d).

The fluorescent intensity in the 3D-EEM spectra could also apply to quantify the EPS concentration [29]. The fluorescence intensities of EPS extracted by boric acid buffer, phosphate buffer, and Tris-HCl buffer methods were greater at peak A and peak B than those of EPS extracted by 0.05% NaCl (*w*/*v*) solution (Table 3), and the fluorescence intensities of EPS extracted by boric acid buffer and Tris-HCl were greater than that of phosphate buffer, which were consistent with the change in protein content (Figure 4). As shown in Table 3, the intensities of peak A and peak B in the NaOH-extracted EPS were 45.07 and 48.15 au, while the intensities of peak A and peak B in phosphate buffer, boric acid buffer, and Tris-HCl buffer extracted EPS were 510.30 and 148.29 au; 348.04 and 625.15 au; and 197.12 and 505.66 au, respectively.

A new fluorescence peak D (tyrosine-like substances) of EPS was detected after NaOH (pH = 10) treatment. This may be caused by the rupture of algal cells and the release of intracellular proteins caused by the NaOH method. Blue shifts were also observed in NaOH-extracted EPS. The interaction between EPS and alkalinity could induce a chemical static fluorescence quenching process, leading to strength reduction and position shift of peaks [30]. This suggests that the tyrosine-like, tryptophan-like, and humic acid-like substances in EPS have stronger interactions with the pH of the solution compared to the buffered solution. Similarly, Kuzhiumparambil et al. found that negatively charged functional groups of EPS can bind to metal hydroxides; tyrosine-like and tryptophan-like substances are the main components of the reaction with heavy metals [31]. Therefore, the pH of the extraction solution should be carefully considered when extracting EPS.

### 3.5. The Particle Size Distribution (PSD) and Morphology of Microcystis

**Figure 5:**
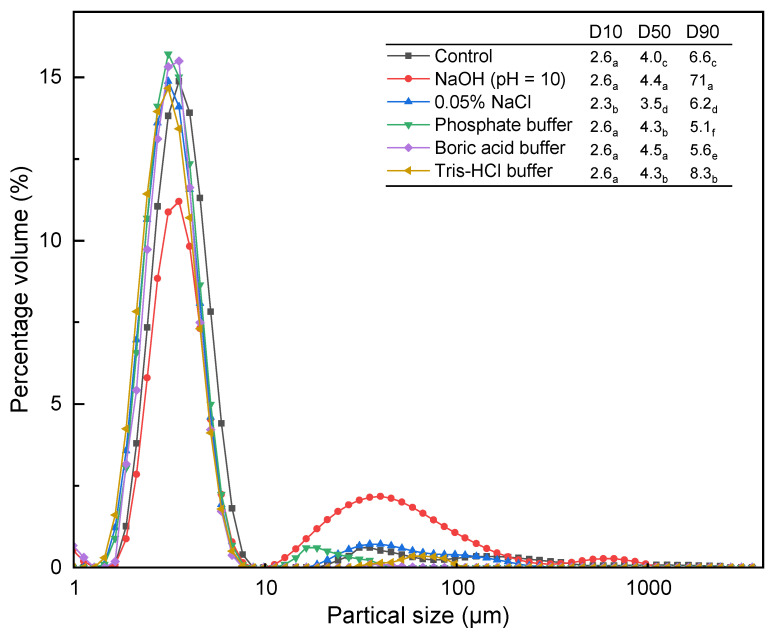
PSD of *Microcystis* after extraction of extracellular polymeric substances by different methods. Dx is particle size (μm), where x% of the total mass of particles is smaller than it. D10, D50, and D90 represent the minimum, median, and maximum particle size in the suspension, respectively. Different letters indicate significance between treatments (*p* < 0.05).

The PSD of *Microcystis* treated with buffer solution and 0.05% NaCl solution did not change significantly, except for a slight decrease in particle size (Figure 5). The result is consistent with the analysis results of FCM. However, the PSD results did not show the presence of cellular debris found during FCM in the NaOH (pH = 10) test. This may be due to the lower sensitivity of the laser diffraction particle size analyzer for smaller size particles [32]. Compared to other treatment methods, treatment with NaOH solution (pH 10) resulted in an approximately 4% reduction in the proportion of *Microcystis* cells within the 1–10 µm size range. Moreover, laser particle size analysis detected *Microcystis* aggregates within the 10–100 µm size range exhibiting a maximum dimension of 976 µm, significantly larger than those observed with alternative extraction methods. This indicates that rupture of the algal cells occurred under strong alkaline conditions, releasing intracellular material. This is consistent with the high CL and FCM analysis of NaOH (pH = 10) treatment. During the extraction, the metal ions contained in EPS and intracellular material of *Microcystis*, such as Ca^2+^, Fe^3+^ and Mg^2+^, were released. At higher pH, these metal ions can form metal hydroxide precipitates with a positive charge, which could attract the negatively charged EPS from algal cells [33]. This would result in compression of the electric double layer and thus the destabilization and finally flocculation of microalgal cells [1]. The mechanism has been applied to microalgae harvesting in the biofuel field [34,35].

**Figure 6:**
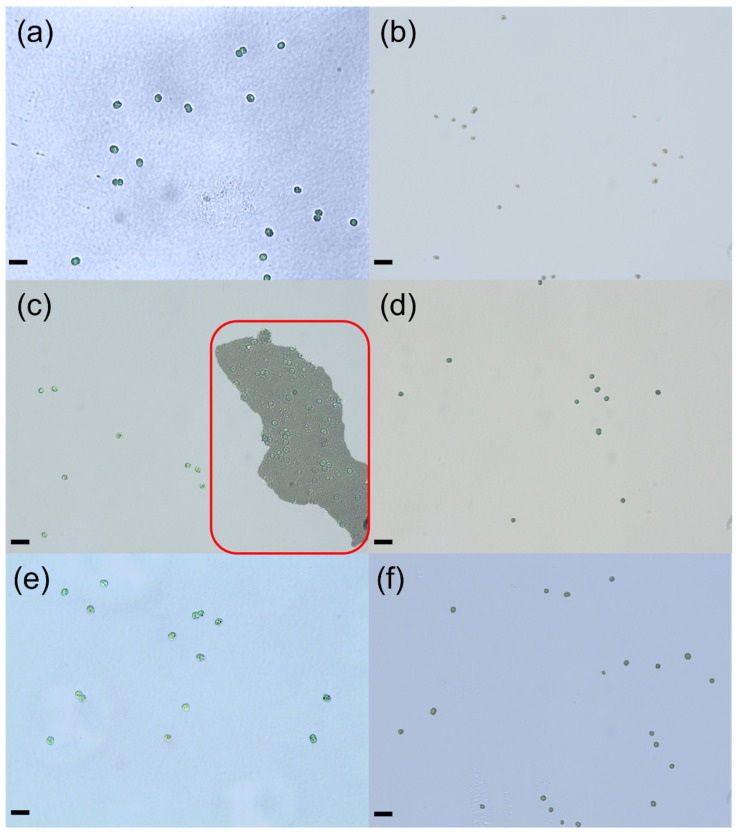
Changes in the morphological characteristics of *Microcystis* cells after the extraction of extracellular polymeric substances by different methods. Control group without treatment (**a**), 0.05% NaCl (*w*/*v*) solution (**b**), NaOH (pH = 10) solution containing an aggregate of *Microcystis* cells in red box (**c**), phosphate buffer (**d**), boric acid buffer (**e**), Tris-HCl buffer (**f**). Scale bars indicate 5 μm.

As shown in Figure 6, cell debris and higher cell transparency were observed under an optical microscope. In particular, the aggregation of *Microcystis* cells could be observed with the NaOH (pH = 10) method (Figure 6c), which is consistent with the results of FCM and PSD analysis. As phosphate and Tris-HCl buffer solution were used for extraction, cell transparency appeared to increase, but almost no cell debris was observed (Figure 6d,f). Similarly, few cellular fragments were observed following treatment with borate buffer solution, and algal cell transparency closely approximated that of the control group (Figure 6a,e). This validates that thermal boric acid treatment could obtain lower cellular lysis.

### 3.6. Extraction Efficiency of Different Methods and Conditions

Table 4 lists the extraction efficiency parameters of the different methods. The proportion of extracted B-EPS is represented by the parameter *φ*. The lowest φ value of 0.03 ± 0.01 was obtained by NaOH treatment, which was the lowest among the five thermal treatment methods. The severe cell lysis during NaOH treatment decreased the calculated *φ* value, indicating that the B-EPS extracted through the treatment were relatively highly contaminated by intracellular organic compounds. For the 0.05% NaCl (*w*/*v*) solution, the low *φ* value was attributed to the low yield of polysaccharides and proteins. This is possibly due to coaggregation of polysaccharides and proteins, which reduced the measurement output and accelerated protein degradation at higher temperatures, leading to a faster decrease in protein than polysaccharide content in *Microcystis*. For the thermal buffer solution treatment, the highest φ value was obtained by the Tris-HCl buffer method, followed by the boric acid buffer method, and the lowest was obtained by the phosphate buffer method, which were 0.94 ± 0.04, 0.62 ± 0.02, and 0.29 ± 0.02, respectively. The results showed that the Tris-HCl buffer method extracted most of the B-EPS under conditions of 60 °C and 30 min. The elevated *φ* value primarily stems from (i) higher EPS extraction yield during isolation and (ii) diminished algal cell lysis during processing, collectively contributing to lower nucleic acid content in the extracts.

The extraction efficiency ε was quantitatively evaluated by integrating the contents of extracted B-EPS and the organic matter content. For NaOH (pH = 10), the efficiency *ε* value (0.03 ± 0.01) was lower than that of other methods, which was caused by the highest cell lysis (*α* = 0.30), resulting in the release of up to 0.52 ± 0.11 mg L^−1^ nucleic acids. These results were contrary to the conclusions of Liu’s study, which found that the NaOH (pH = 10) method enhanced fluorescence intensity of EPSs and considered NaOH extraction to be better than the extraction method [14]. However, in our study, NaOH treatment caused high cell lysis and intracellular substance release. This is consistent with the results of Duan et al., who believed that the NaOH extraction method could be used to extract intracellular substances (such as phycocyanin) from cyanobacteria [12]. The 0.05% NaCl method extracted less B-EPS, resulting in a lower *ε* value of 2.13 ± 0.19, although the proportion of ruptured cells was only 0.098. Some researchers increased the NaCl concentration to increase the osmotic pressure of the extraction solution to maintain cell integrity [29]. Notably, the EPS extracted by this method should consider the influence of NaCl in subsequent analyses and should be removed if necessary, such as dialysis [36]. The ε values of the three buffer extraction methods are relatively higher than other extraction methods, among which the boric acid buffer extraction method has the highest ε value of 11.06 ± 1.13, with only 6.9% of cells ruptured and 62% of B-EPS separated from cells. The Tris-HCl buffer extraction method has a slightly lower ε value (9.7 ± 0.81) than the boric acid buffer extraction method, which is due to the higher cell rupture rate, with α = 0.12 ± 0.02. The extraction conditions with higher ε values are relatively mild, indicating that moderate conditions are more suitable for extracting EPS [13].

## 4. Conclusions

In this study, we established an evaluation system for algal EPS thermal extraction methods integrating extraction yield, cell integrity, and EPS chemical properties. The optimal operating parameters in buffer (boric, phosphate, Tris-HCl) were compared to two widely employed methods in previous studies. The results showed that the thermal buffer solution with NaCl (0.5% *w*/*v*) could achieve the highest EPS yield and the lowest cell lysis and the optimal extraction efficiency ε, which followed the order of boric acid buffer treatment > Tris-HCl buffer treatment > phosphate buffer treatment (3.90 ± 0.28, 55 °C, 30 min) > 0.05% NaCl (*w*/*v*) solution > NaOH (pH = 10) treatment. The thermal buffer extraction method with increased osmotic pressure can enhance the extraction efficiency of EPS from *Microcystis*, as stable pH and high salt concentration. Additionally, the EPS extraction efficiency evaluation model in this study can be applied to other microorganisms for developing extraction processes and quantitatively assessing EPS extraction efficiency.

Moreover, an effective thermal EPS extraction process can be developed for other microalgae through using boric acid buffer and the combination of the evaluation system for algal EPS extraction methods in this study. Meanwhile, further studies and in-depth works should be considered. For example, establishing an EPS extraction method database.

## Figures and Tables

**Table 1:** Maximum fields and extraction conditions of B-EPS extracted from *Microcystis* via thermal buffers.

Extraction Methods	Extraction Conditions	Polysaccharides (mg L^−1^)	Proteins (mg L^−1^)	TOC (mg L^−1^)
Phosphate buffer	55 °C, 30 min	0.73 ± 0.08	0.68 ± 0.08	1.55 ± 0.10
Boric acid buffer	55 °C, 30 min	1.33 ± 0.11	0.89 ± 0.07	2.81 ± 0.15
Tris-HCl buffer	60 °C, 30 min	2.98 ± 0.08	2.28 ± 0.18	5.6 ± 0.064

**Table 2:** FSC, FITC, and cell lysis (CL) of *Microcystis* via different thermal treatments.

	FSC			FITC			CL (%)
	Events in Gate (%)		Signal Intensity	Events in Gate (%)		Fluorescent Signal	
	P1	P2	Mean ± SD (10^5^)	Q1	Q2	Mean ± SD (10^5^)	
Control	5.4	94.6	1.5 ± 0.25	2.6	97.4	0.06 ± 0.001	2.1
Phosphate buffer	6.6	93.4	1.3 ± 0.02	3.0	97.0	0.05 ± 0.001	9.3
Boric acid buffer	8.1	91.9	1.2 ± 0.02	9.8	90.2	0.05 ± 0.001	6.9
Tris-HCl buffer	5.7	94.3	1.1 ± 0.08	1.2	98.8	0.04 ± 0.001	12.0
0.05% NaCl	30.5	69.5	0.7 ± 0.01	1.4	98.6	0.04 ± 0.005	9.8
NaOH (pH = 10)	49.8	50.2	0.7 ± 0.04	16.5	83.5	0.03 ± 0.01	30.4

Note: FSC refers to the signal of forward scatter. FITC represents the fluorescence signal at 518–548 nm. CL is the ratio of broken algae cells. The P1 and P2 gates indicate the percentages of cell debris and intact cells, respectively. The Q1 and Q2 gates indicate the percentages of cells or cell debris without FITC fluorescence and cells with FITC fluorescence, respectively. A total of 20,000 events were recorded for each sample, and the results are reported as mean ± standard deviation (SD).

**Table 3:** The composition changes of the EPS extracted by different methods indicated by the 3D-EEM.

Methods	Peak A		Peak B		Peak C		Peak D	
	Ex/Em	Intensity	Ex/Em	Intensity	Ex/Em	Intensity	Ex/Em	Intensity
Control	280/335	66.38	250/450	22.32	355/455	14.45	/	/
0.05% NaCl	280/335	206.50	225/335	144.05	355/455	53.67	230/330	138.62
NaOH (pH = 10)	280/340	45.07	225/340	48.15	360/455	30.46	230/330	81.09
Phosphate buffer	280/335	510.30	225/330	148.29	/	/	/	/
Boric acid buffer	280/335	348.04	250/450	625.15	355/455	296.75	/	/
Tris-HCl buffer	280/335	197.12	250/450	505.66	355/455	283.26	/	/

**Table 4:** Optimal conditions and corresponding extraction efficiency evaluation parameters of tested methods for *Microcystis*.

Methods	Conditions	Calculated Parameters		
		α	φ	ε
Control	–	0.02 ± 0.01 ^a^	0.04 ± 0.01 ^a^	2.56 ± 0.22 ^b^
0.05% NaCl	60 °C, 30 min	0.098 ± 0.01 ^a^	0.17 ± 0.02 ^a^	2.13 ± 0.19 ^b^
NaOH (pH = 10)	45 °C, 4 h	0.30 ± 0.02 ^b^	0.03 ± 0.01 ^a^	0.14 ± 0.01 ^a^
Phosphate buffer	55 °C, 30 min	0.093 ± 0.01 ^a^	0.29 ± 0.02 ^a^	3.90 ± 0.28 ^b^
Boric acid buffer	55 °C, 30 min	0.069 ± 0.01 ^a^	0.62 ± 0.02 ^b^	11.06 ± 1.13 ^c^
Tris-HCl buffer	60 °C, 30 min	0.12 ± 0.02 ^a^	0.94 ± 0.04 ^b^	9.7 ± 0.81 ^c^

Data are shown as the mean ± standard deviation (SD) (n = 3). Different letters indicate significance between different treatments (*p* < 0.05).

**Figure A1:**
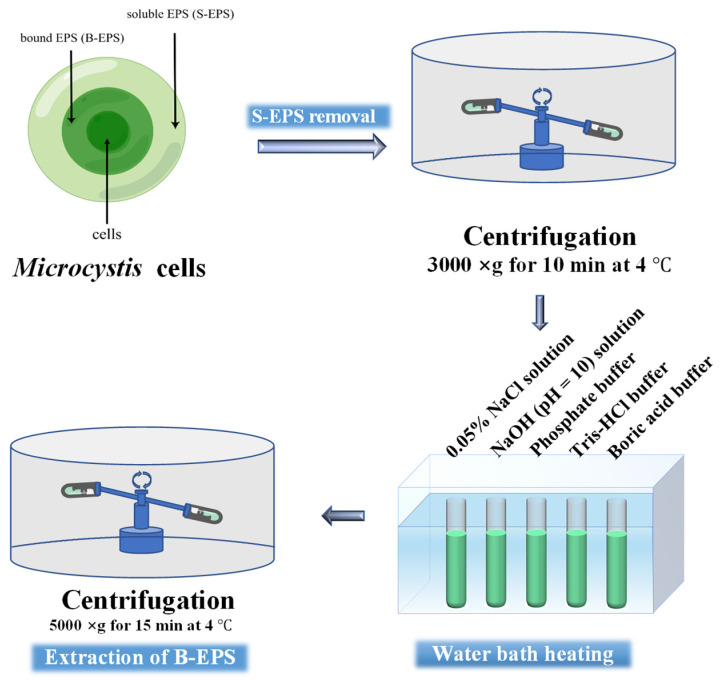
B-EPS extraction process from *Microcystis*.

## Data Availability

The original contributions presented in this study are included in the article. Further inquiries can be directed to the corresponding author.

## Appendix A. EPS Extraction

The extraction method of S-EPS was relatively fixed, that is, centrifugation, but the extraction method of bound EPS was diversified. Therefore, the extraction method of bound EPS was the focus of attention, and EPS referred to bound EPS in this study. To compare the different EPS extraction methods, the widely used thermal methods were selected in this study: 0.05% NaCl (*w*/*v*) method and NaOH (pH = 10) method.

Cultured algal cells in logarithmic growth phase were collected by centrifugation (3000× *g*, 4 °C, 10 min) and then resuspended in extracts of 0.05% NaCl (*w*/*v*) solution and NaOH solution (pH = 10) at the same cell concentration of 4.5 × 10^6^ cells mL^−1^. For the 0.05% NaCl (*w*/*v*) method, algae cell suspensions were heated for 4 h in a 45 °C water bath. For NaOH (pH = 10) solution, the suspensions were heated for 30 min in a 60 °C water bath (Figure A1).

To improve the EPS yield and reduce cell rupture during the extraction process, in this study, we modified the commonly used thermal extraction method by trying to maintain pH stability during the extraction process using buffer solutions (borate buffer, phosphate buffer, Tris-HCl buffer) and adding NaCl (0.5% *w*/*v*) to maintain the stability of algal cell osmolarity. The specific operation is shown below. The pH values of boric acid buffer, phosphate buffer, and Tris-HCl buffer were adjusted to 8.4. NaCl (0.5% *w*/*v*) solution was added to buffers to sustain stable cellular osmolarity during the process of EPS extraction. Cultured algal cells in logarithmic growth phase were collected by centrifugation (3000× *g*, 4 °C, 10 min) and then resuspended in buffers at a cell density of 4.5 × 10^6^ cells mL^−1^. The water bath was utilized for the thermal treatment of algae cell suspensions at the temperature range of 45 to 65 °C, and each temperature gradient was processed for 15, 20, 25, and 30 min. The algae suspensions maintained at 25 °C were control groups. After the aforementioned treatment, the algal cells were harvested by centrifugation (5000× *g*, 4 °C, 15 min), and the supernatants were filtered and stored at 4 °C until analysis. All experimental treatments were performed in triplicate. Simultaneously, the collected algal cells were resuspended in fresh medium to evaluate their cellular integrity and morphology using flow cytometry (FCM) and the optical microscope.
